# Physical activity level in elderly assisted in an outpatient of comprehensive health care

**DOI:** 10.1186/1758-5996-7-S1-A226

**Published:** 2015-11-11

**Authors:** Ana Tamires Jardim, Joelma Ximenes Prado Teixeira Nascimento, Ana Tamires Jardim, Joelma Ximenes Prado Teixeira Nascimento

**Affiliations:** 1 Universidade Ceuma, São Luis, Brazil

## Background

Old age brings many changes and deleterious effects with advancing age, to minimize these declines regular physical activity has a positive effect on prevention and promotion of health of the elderly.

## Objective

To identify physical activity level in elderly assisted in an outpatient of comprehensive health care.

## Materials and methods

Cross-sectional study with 42 elderly treated at a clinic from a private university in São Luís-MA, conducted between the months from March to May 2015. We collected data on sociodemographic, economic, clinical and nutritional characteristics, anthropometric measurements and physical activity through the IPAQ (International Physical Activity Questionnaire). It was used the procedures of descriptive statistics and measures of association where the level of significance adopted was p <0.05. Data were analyzed using program Stata 12.0®.

## Results

The distribution between age groups showed a higher proportion of elderly between 60 and 69 yrs. (54.76%), 78.57% had only elementary education, married (57.14%), retired (52.38%) and received up to 3 minimum wages (76.16%). Regarding physical activity, 80.95% were inactive, being more pronounced with increasing age (42.86%), associated with this, overweight diagnosis (13.8%), affected by hypertension (22,7%), large medication use (47.62%) and risk for cardiovascular diseases (23.5%).

## Conclusion

The findings of the study suggest the need for intervention and encouraging physical activity associated with healthy living habits, considering besides the health aspects of older, the reality of this population.

**Figure 1:**
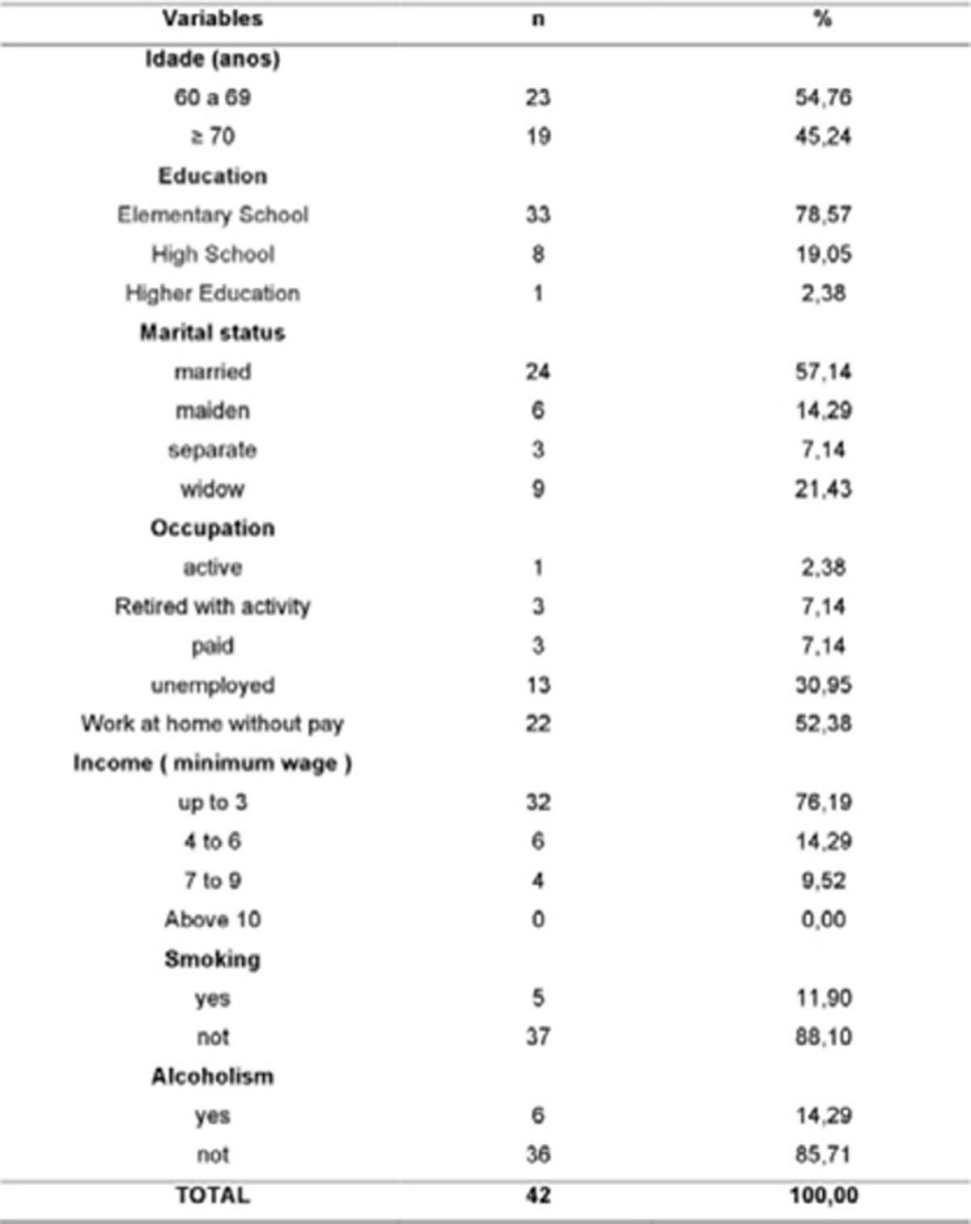
Socioeconomic, demographic and behavioral characteristics of elderly assistant in an outpatient of comprehensive health caer. Sao Luis, 2015

**Figure 2:**
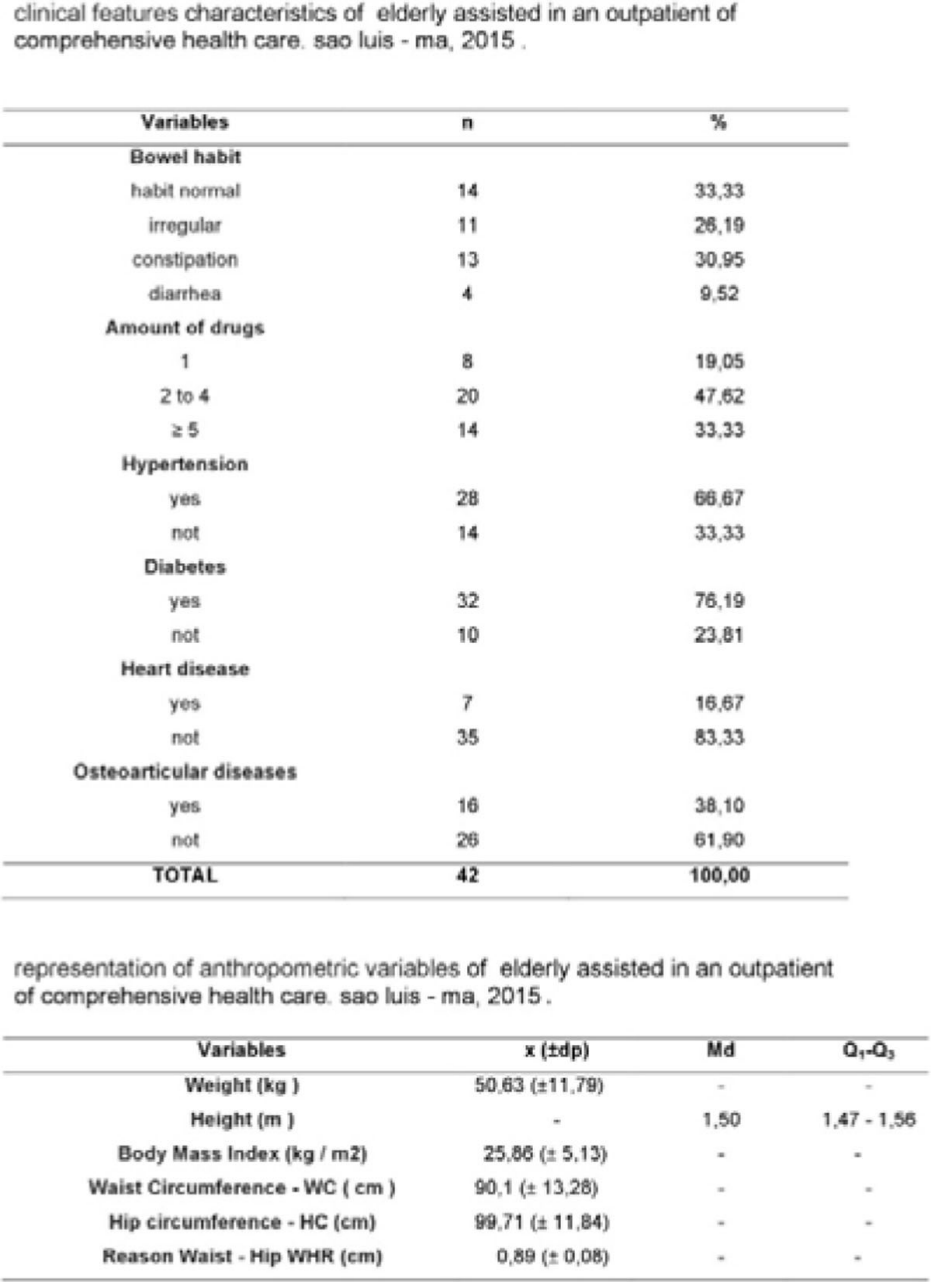
Clinical feature characteristics of elderly assisted in an outpatient of comprehensive health care. Sao Luis – Ma, 2015.

**Figure 3:**
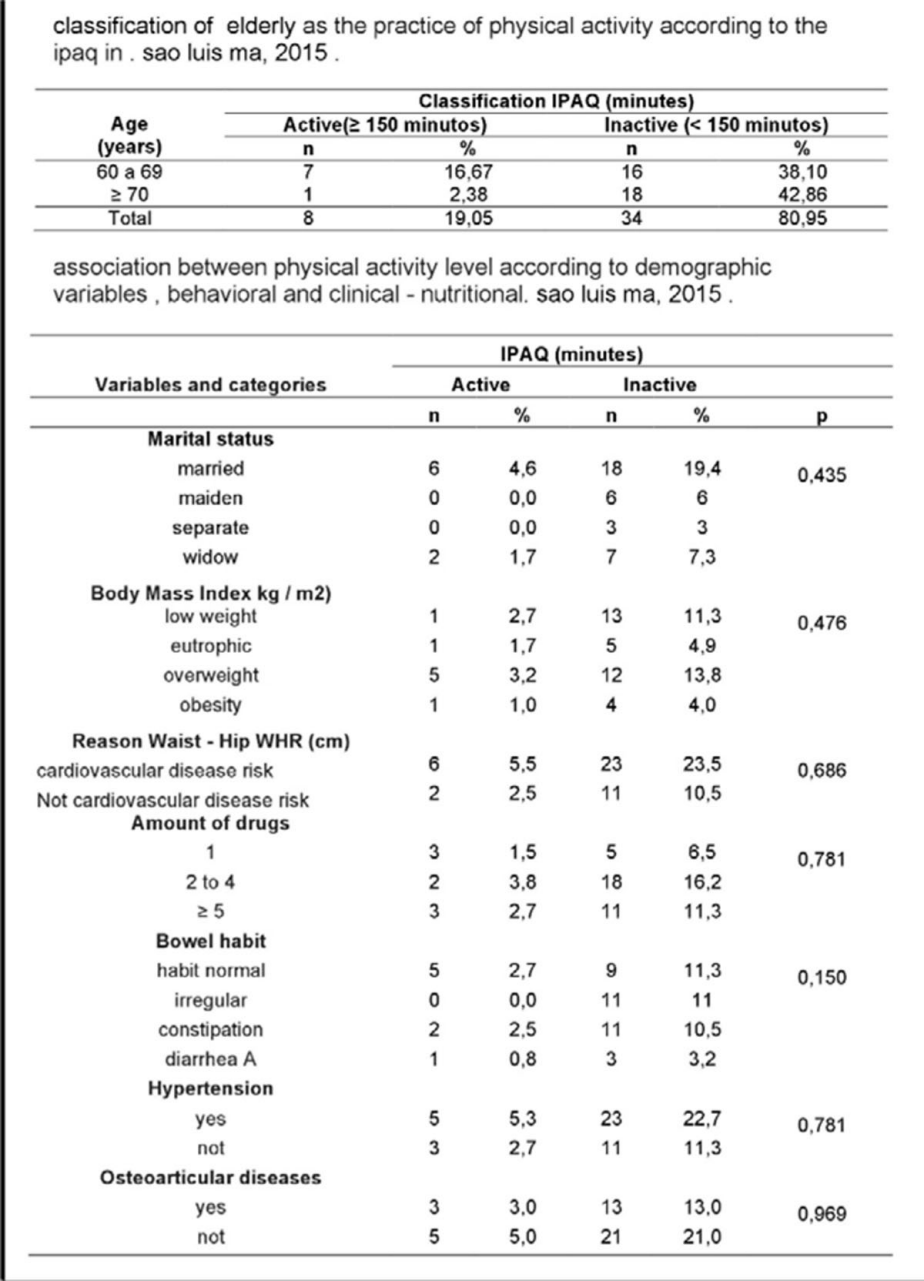
Classification of elderly as the practice of physical activity according to the IPAQ in Sao Luis Ma, 2015.

